# Remarkable improvement in drilling fluid properties with graphitic-carbon nitride for enhanced wellbore stability

**DOI:** 10.1016/j.heliyon.2024.e41237

**Published:** 2024-12-14

**Authors:** Anwar Ahmed, Erum Pervaiz, Iftikhar Ahmed, Tayyaba Noor

**Affiliations:** a Department of Chemical Engineering, School of Chemical and Materials Engineering (SCME), National University of Sciences & Technology (NUST), Sector H-12, Islamabad, 44000, Pakistan; b Environment and Public Health Department, College of Health Sciences, Abu Dhabi University, Abu Dhabi, 59911, United Arab Emirates

**Keywords:** Drilling fluids, Graphitic carbon nitride, Permeability plugging, Shale stability, BET isotherms

## Abstract

This study examines the viability of using graphitic-Carbon Nitride (g-C_3_N_4_) nanomaterial as shale stabilizer drilling fluid additive having applications in the oil and gas wells drilling. Shale stability is important especially when drilling horizontal and extended reach wells with water-based muds (WBM) to tap unconventional reservoirs namely shale oil and shale gas. For this study, the g-C_3_N_4_ nanomaterial was produced by melamine pyrolysis, and characterized by X-Ray Diffraction, Scanning Electron Microscopy and Fourier Transform Infrared spectroscopy techniques. The developed g-C_3_N_4_ was used to formulate the WBM and its impact on the formulated mud system's rheological and filtration control characteristics as well as on shale stability was examined. In comparison to the base mud, the treated mud showed lower Fluid Loss (FL) and higher thermal stability. FL was reduced by 41.8 % and 68 % under Before Hot Rolling (BHR) and After Hot Rolling (AHR) conditions, respectively, with a maximum cake thickness of 1 mm. The Yield Point was improved by 52 % and 66 % under BHR and AHR conditions, respectively. The increase in Plastic Viscosity, and Apparent Viscosity was 23.8 %, and 38 %, respectively. Shale recovery was 99.6 % in g-C_3_N_4_ treated fluid compared to 88 % in the base fluid. The treated shale Brunauer-Emmett-Teller (BET) surface area and the pore volume were significantly reduced compared to the pure shale, indicating significant plugging of shale nano- and micro-pores. The BET surface area of the g-C_3_N_4_ treated shale sample was 0.0405 m^2^/g compared to pure shale sample's surface area 0.3501 m^2^/g. Correspondingly, the pore volume of treated shale was 0.000029 cm^3^/g compared to the pure shale sample's pore volume 0.000911 cm^3^/g. Therefore, based on the experimental results obtained, it is inferred that the developed g-C_3_N_4_ nanomaterial has potential applications in WBM systems for drilling long shale sections.

## 1 Introduction

The production from known conventional hydrocarbon reservoirs is estimated to peak by 2040 [1]. However, as the world's energy demand is increasing, there is shift towards the exploration of unconventional hydrocarbon reservoirs especially the shale oil and shale gas [2]. However, drilling of shale formation with WBM, which is being preferred now a day due to environmental reasons, poses certain shale instability problems. Shale instability problems become further challenging when drilling multilateral and extended reach wells to tap longer shale sections. Drilling fluids laden with appropriate additives such as nanomaterials are helpful in overcoming the shale instability problems while drilling deep and extended reach wells [3].

Drilling fluids or muds are chemical mixtures that are essential to the drilling industry [4,5]. Drilling fluids are normally classified in three types, namely, Water Based Drilling Fluids, Oil Based Drilling Fluids and Synthetic Based Drilling Fluids. Although Oil Based and Synthetic Based Drilling Fluids minimize shale instability issues to great extent, however, due to environmental reasons, Water Based Drilling Fluids are preferred in most wells now a day. Chemicals that are added in drilling fluids result in specific fluid characteristics such as density, viscosity and fluid loss suitable for achieving the drilling objectives safely [6].

Specialized additives, such as nanomaterials, are used to overcome the anticipated downhole problems such as shale instability or downhole losses [7]. The invention of the unique characteristics of nanomaterials and their incorporation in drilling muds have benefited in achieving the improved filtration, better formation stability, enhanced cuttings removal and lower friction; the key parameters required for the safer drilling of shale sections in extended reach wells. The combined effect of drilling fluid methodologies and nanomaterial studies in recent years has resulted in enhancing the overall drilling efficiency [8,9]. The optimization of drilling parameters not only helps to achieve the drilling targets technically, but also saves additional cost [10]. The nanomaterials have demonstrated a favorable influence on oil and gas exploration, both in terms of rate of penetration and trouble free drilling, assuring safer and smooth drilling operations [11]. Considerable results have been achieved with nanostructures of up to 100 nm size, with some key studies demonstrating enhanced drilling fluid rheological performance [12,13].

Because of their thin film forming characteristics, nanomaterial-based drilling fluids depict improved lubricious characteristics minimizing friction between drill string and well bore [14]. In addition to generating thin lubricant layers between pipe and the wall, tiny nanomaterial particles between the wall of drill pipe and boreholes also act as solid lubricants providing additional lubricity and making drilling faster [15]. Much research published on the creation of nanoparticles (NPs) [16] shows considerable changes in the features of mud systems. The inclusion of nanomaterials allows for the creation of innovative additives and drilling fluid systems that can handle higher stress and heat, decrease formation damage by reducing fluid loss, improve borehole strength, and enhance drilling fluid resistance to salts and calcium hardness [17].

Salt effects have been studied by different researchers in connection with shale stability. In recent works, the salt effects have been studied with drilling fluids containing the nanomaterials. Thus, J.O. Oseh et al. investigated the effect of sodium dodecyl sulphate-treated nanohydroxyapatite (nHAp/SDS) on shale stability using it with Potassium Chloride (KCl)-containing WBM. They conducted the dispersion tests and compared these with nano-silica, inferring the nHAP/SDS performance better than the nano-silica [18]. Lalji and coworkers studied the effect of using Graphene Oxide nanoparticles in a WBM formulation and demonstrated its effectiveness in minimizing shale swelling compared to the base mud [19]. In another study, Yahya et al. demonstrated the effectiveness of graphene nanoplatelets using these in WBM and showing improved lubricity, rheology, filtration and inhibition properties compared to the base mud [20].

Jeffrey et al. investigated the effect of Hydroxyapatite nanoparticles (Nano-HAp) on bentonite-based mud (BN-WBM) rheology and filtration properties in the presence of varying concentrations of sodium chloride (NaCl) and calcium chloride (CaCl_2_) from 77 to 410 °F. They observed reduction in viscosity and fluid loss when 0.5 % Nano-HAp was added in the BN-WBM due to the adsorption of Nano-HAp on the bentonite platelets minimizing direct salt interaction with it. The reduction in both the viscosity and the fluid loss parameters was slightly higher in the CaCl_2_ contaminated mud compared to NaCl contaminated mud, thus indicating the salt tolerance of the BN-WBM under high temperature conditions [21]. In another similar study Alireza Rezaei et al. investigated the effect of Magnetic Oxide (Fe_3_O_4_) NPs on rheological and filtration properties of the bentonite-based drilling fluids in the presence of NaCl, CaCl_2_ and Magnesium Chloride (MgCl_2_) salts. Although salts weaken the rheological and filtration properties of the base fluids, Fe_3_O_4_ NPs sustain the same properties of the salt treated bentonite-based fluid, proving nano-Fe_3_O_4_ to be a suitable additive for improving the salty WBMs performance [22].

Nanomaterials are being considered as an effective tool to address downhole drilling problems such as formation damage, hole cleaning constraints, lost circulation, wellbore instability and drill string sticking. Many NPs including titanium dioxide, silica and zinc oxide have been studied in this regard by examining their impact on the rheological properties and shale stability [23]. Such studies have indicated that nano-silica improves mud cake density consequently reducing the filtration loss, decreases shale permeability minimizing pressure transmission as well as improving mud lubricity and rheology [24]. Colloidal and dispersion stability is another requisite feature of drilling fluids to perform their optimum function. Since nanomaterials exist as colloids in the fluid form, it is important to maintain their colloidal and dispersion stability in the fluids to get their optimum function.

Nanomaterials also improve the thermal and mechanical stability of WBMs. In conventional WBMs, biopolymers, such as Xanthan Gum and Starch, being the environmentally friendly polymers, are used to preserve fluid rheology, filtration, viscosity, and other features [25,26]. However, under extreme circumstances, for example at High Pressure High Temperature (HPHT) wells [27], these biopolymers degrade rapidly and lose their characteristics [28,29]. Resultantly, mud systems require stable ingredients to withstand such harsh conditions. Because of their great thermal and mechanical resilience under HPHT conditions, nanomaterials when added to drilling fluids extend thermal and mechanical stability of muds to higher temperature and pressure levels.

In addition to improving the fluid rheology and filtrate control, nanomaterials also show significant shale plugging properties as demonstrated by BET studies which are used to determine the surface area, pore size and pore volume of shales to examine the plugging phenomenon during oil well drilling [30,31]. Thus, Wang et al. measured the surface properties of the amino-montmorillonite in connection with their studies on the material being used for drilling applications [32]. Wuquan Li and co-researchers studied the plugging of shale with styrene butadiene resin/nano-SiO_2_ (SBR/nano-SiO_2_) composite using BET surface studies technique and found SBR/nano-SiO_2_ an effective shale plugging agent [33].

Carbon-based NPs are the extensively used nanomaterials, having wide range of applications in the industry [34]. It is the most adaptable material ever created, with the ability to combine with other chemicals and give a wide range of structures, leading to great industrial progress [35,36]. In the current study, based on its characteristics, g-C_3_N_4_, which is also a carbon-based nanomaterial, has been identified to improve the rheological and filtration control properties, enhance the thermal and mechanical stability and minimize shale instability problems when used in the drilling fluids. The g-C_3_N_4_ structure is composed of a hexagonal unit in which carbon atoms are joined together by covalent bonds. The single covalent bonds are chemically very strong bonds with a higher binding energy that can only be broken by a massive amount of energy. As a result, g-C_3_N_4_ has a high thermal resistance [37,38]. The g-C_3_N_4_ nanosheets have a large specific surface area, may produce a thinner durable film via the connecting channel in mud system, increasing the qualities of the fluid with lesser quantity of the nanomaterial [39]. The g-C_3_N_4_ when incorporated in the drilling fluid, is anticipated to provide additional filtration control by sealing the shale nanopores and minimizing filtrate invasion into the formation. This process decreases the nanoscale hole propagation in the formation, therefore, enhancing the quality of mud cake [40]. Additionally, g-C_3_N_4_ like graphene can enter the pores of tubulars, where they would undergo crystallization under intense pressure and making a lubricating, protective coating that would reduce the torque for water-based mud [41].

Table 1 compares the shale instability reasons and related downhole conditions which lead to wellbore instability and compares the g-C_3_N_4_ properties making it suitable to address the shale instability reasons and remaining stable under the downhole conditions [42,43].

**Table 1:** Shale Characteristics and Suitability of g-C_3_N_4_ for its Plugging.

Characteristics of Shale	g-C_3_N_4_ Properties
• Water sensitive having swelling and dispersive nature • Requires some stabilizing phenomenon (such as Ion Exchange, Surface Coating, Limiting Pore Pressure Transmission, Permeability Plugging) • Pore size in nanoscale range • Wellbore strengthening requirement • Higher bottom hole temperature in deeper horizons • Shales show time dependent instability • Shales depict a varying degree of porosity and permeability	• Sustainable material; made of Carbon & Nitrogen only • Water insoluble, Inert material • Wider specific surface area, suitable for adsorption • Robustness of structure • Large linear elasticity (resilience) • Environment friendly material • Thermally stable to 1200 °F • Has applications in emulsion stabilization • g-C_3_N_4_ and its hybrids can address most of shale instability issues

The plan in this research is to develop and explore the usage of g-C_3_N_4_ in drilling fluids and exploit its large surface area and enhanced thermal, mechanical and adsorption properties due to its two dimensional (2D) sheet like structure for improving drilling fluid properties [44]. g-C_3_N_4_ is a greener product, compared to simple graphite which has been traditionally used in the drilling fluids. Compared to graphite which is pure carbon, some of the carbon in g-C_3_N_4_ has been replaced by nitrogen thus reducing the carbon footprint. The study would lead to the application of g-C_3_N_4_ in drilling fluids to be used for drilling directional and extended reach wells containing long shale sections as well as to tap the unconventional hydrocarbon reservoirs such as shale oil and shale gas. As per our knowledge g-C_3_N_4_ in its bare form has not been used earlier in drilling fluids.

## 2 Material and methods

Melamine (≥99 %), deionized water and ethanol were sourced from Sigma-Aldrich, USA. All the chemicals used in this research were of analytical grade and in their original form.

### 2.1 Synthesis of g-C_3_N_4_

**Fig. 1:**
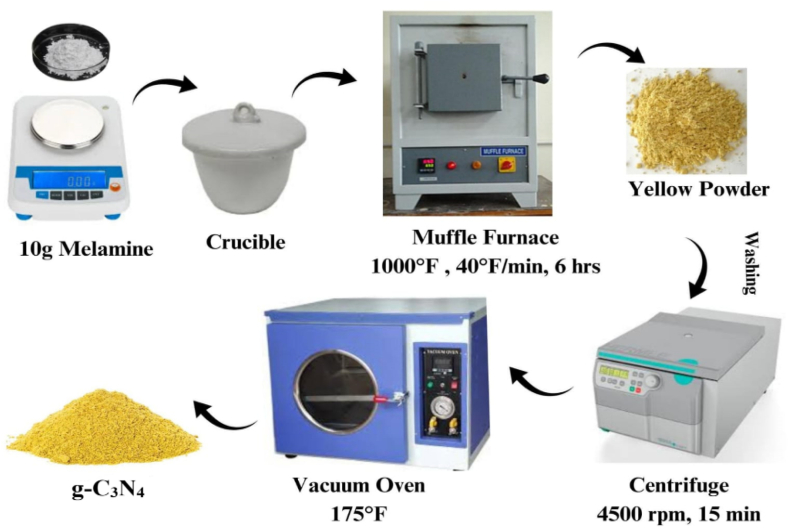
Schematic Representation of g-C_3_N_4_ synthesis.

A process reported in the literature was used for the synthesis of the bulk g-C_3_N_4_ [45]. An alumina crucible containing 10 g of melamine was placed in a muffle furnace and heated to 1000 °F over the course of 6 h at an acceleration rate of 40 °F per minute. Following that, it was kept at ambient temperature, namely 85 °F. As a result of calcination, a yellow-coloured cluster developed, which was further ground in a mortar and pestle to a fine powder representing the raw g-C_3_N_4_. This yellow powder was further given 2–3 washings with distilled water and ethanol each, using a laboratory centrifuge at 4500 rpm for 15 min each time. The residue was extracted in a Petri dish, dried in vacuum oven at 175 °F for 6 h. The dry residue was finely ground to get the final product which was used for characterization and further analysis. The schematic representation of the g-C_3_N_4_ synthesis is shown in Fig. 1 below.

### 2.2 Characterization

#### 2.2.1 X-Ray Diffraction (XRD)

X-Ray Diffractometer (STOE - Germany) was used to analyze the developed g-C_3_N_4_. The current and voltage of the X-Ray source were 40 mA and 40 kV, respectively. A 2θ range of 10–80° was used to scan the sample. The crystallite size of g-C_3_N_4_ was calculated using the Scherrer equation expressed below as Equation (1):

(1) *L=Kλ/β*.cosθ

Where L is the nano crystallite size, K is the Scherrer constant and a dimensionless quantity, *λ* is X-Ray wavelength in nm, *β* in radian is full width at half maximum of peaks with θ indicating the scan range in degrees.

#### 2.2.2 Scanning Electron Microscopy (SEM)

The surface morphology and microstructure of the prepared sample were investigated using a Scanning Electron Microscope (JEOL-JSM-6490A) coupled with Energy Dispersive X-Ray (EDX).

#### 2.2.3 Fourier Transform Infrared (FTIR) spectroscopy

Fourier Transform Infrared Spectroscopy was used to obtain infrared absorption spectrum of the powdered material to identify the present functional groups.

### 2.3 Development of a nanomaterial-based WBM system

#### 2.3.1 Drilling fluid rheological properties and filtration characteristics

In a Hamilton Beach Mixer, 3.0 w/v% KCl, 0.3 w/v% xanthan gum, and 0.6 w/v% pre gelatinized starch were combined to make the base fluid. Then, according to conventional protocols for the testing of drilling muds, the appropriate quantity of nanomaterial was introduced to examine its influence on the different parameters of the drilling fluid. The designed system's rheology parameters and fluid loss control properties were investigated using an OFITE American Petroleum Institute (API) Filter Press and a Fann Viscometer, model 35SA, both from Original Equipment Manufacturers (OEMs) based in Houston, Texas. Equations (2), (3), (4), as given below were used to calculate the rheological parameters in accordance with API recommendations:

(2) Apparent Viscosity (AV) = Ø_600_/2 (mPa-s)

(3) Plastic Viscosity (PV) = Ø_600_ – Ø_300_ (mPa-s)

(4) Yield point (YP) = (Ø_300 –_ PV) (Pa)

Where Ø_300_ and Ø_600_ are the viscometer dial reading at 300 and 600 rpm, respectively.

#### 2.3.2 Apparatus used in the research study for testing drilling fluids properties

.

#### 2.3.3 The drilling mud system's salt resistance

A specified quantity of the produced nanomaterial (0.5 g, 1.0 g, and 2.0 g) and various concentrations of KCl salt were mixed in the base fluid to test the drilling fluid system's ability to tolerate salt. After that, the base fluid was aged for 16 h at 200 °F. Using methods established by the API, the impact of adding salt on the filtration properties and rheological parameters of the system was examined (see. Table 2 and Table 4).

**Table 2:** Apparatus used for Drilling Fluid Testing.

Device	Function
Hamilton Beach/Proctor-Silex, Inc. Model 1G936 (Assembled in USA)	Used for mixing chemicals to prepare drilling fluids
Fann Viscometer Model 35SA (Assembled in USA)	To check the rheology properties of drilling fluids
OFITE High Pressure High Temperature Filter press	To measure the filtrate volume at high pressure and high temperature and the mud cake
OFITE 141-00-C Filter Press (USA)	To measure filtrate volume and cake thickness at 100 psi applied pressure
OFITE 171-85 Permeability Plugging tester, Made in USA	To measure fluid loss at very high pressure and temperature using ceramic disc of 40 μm

#### 2.3.4 Thermal stability of the drilling mud system

The developed formulation's thermal stability was evaluated by ageing it for 16 h at 225 °F in a roller oven. After aging, the drilling fluid's rheological properties and filtration characteristics were tested again using API-recommended protocols.

### 2.4 Shale stability studies with the developed drilling fluid system containing synthesized nanomaterial

#### 2.4.1 Test for shale recovery using synthesized g-C_3_N_4_

The shale samples were pulverized and screened through 20–30 mesh sieves to see how different concentrations of the synthesized nanomaterial affected shale recovery during fluid hot rolling experiments. In an ageing cell, 15 g (Wo) of the shale cuttings were mixed with the drilling fluid for hot rolling at 225 °F for 16 h. The drilling fluid, which contained shale cuttings, was then filtered through a 50-mesh screen, and the cuttings that remained on the sieve were washed away with water. The weight (W_1_) of the sample was measured after drying at 140 °F, and the shale recovery rate (%R) was computed using Equation (5) as given below:

(5) %R = (W_1_/W_O_)∗100

## 3 Results and discussion

### 3.1 Powder X-ray diffraction

**Fig. 2:**
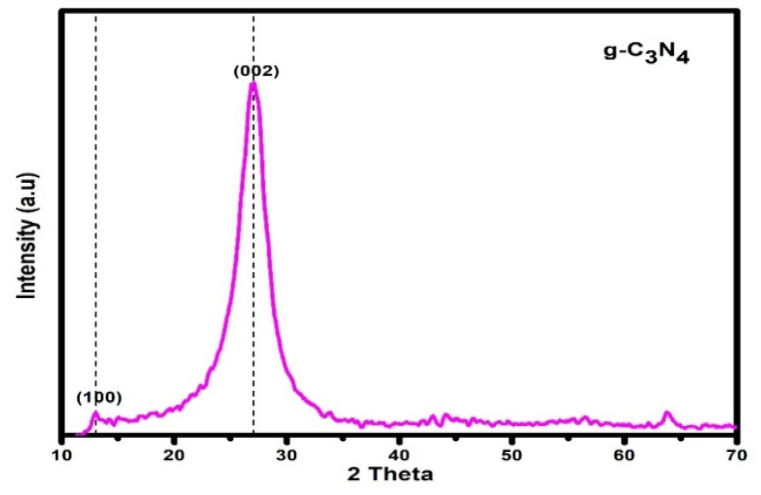
XRD Spectrum of g-C_3_N_4_.

The XRD spectrum of g-C_3_N_4_ is shown in Fig. 2. Two distinct diffraction peaks can be identified on the spectrum. The interlayer stacking of conjugated aromatic planes, indexed as (002) with d-spacing of 0.326 nm, is assigned to the strong peak found at 27.5° and the small peak almost at 12.9° with 0.692 nm d-spacing indexed as (100) is the structural packing motif of tri-s-triazine units [46,47].

### 3.2 FTIR spectroscopy

**Fig. 3:**
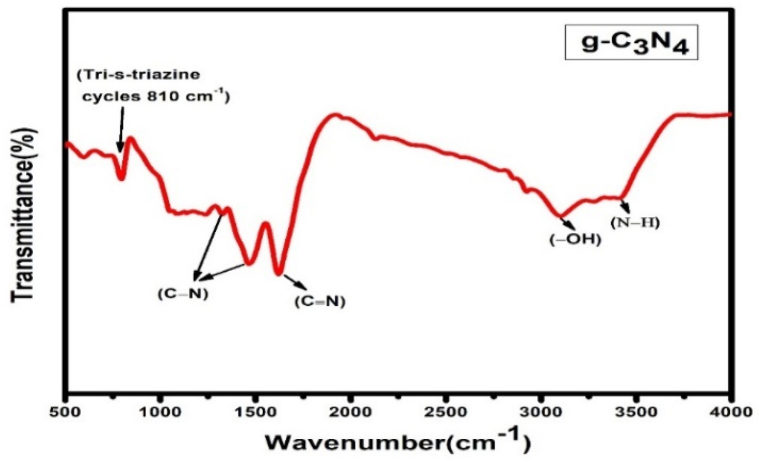
FTIR spectrum of g-C_3_N_4_.

Fig. 3 displays the FTIR spectrum. Three distinguishing bands were discovered in the sampled g-C_3_N_4_ spectrum that were identical to the typical g-C_3_N_4_ structure obtained from the earlier findings [15,48,49]. The strong band between 1200 and 1600 cm^−1^ is connected to the stretching vibration of the C – N and C = N heterocycles [48], whereas the absorption peak at 810 cm^−1^ relates to the distinctive breathing mode of tri-s-triazine cycles [15]. The stretching vibrations of the terminal N – H originating from uncondensed amino groups are considered to be the basis of the large absorption peak in the range of 3000–3500 cm^−1^ [49].

### 3.3 Scanning Electron Microscopy

**Fig. 4:**
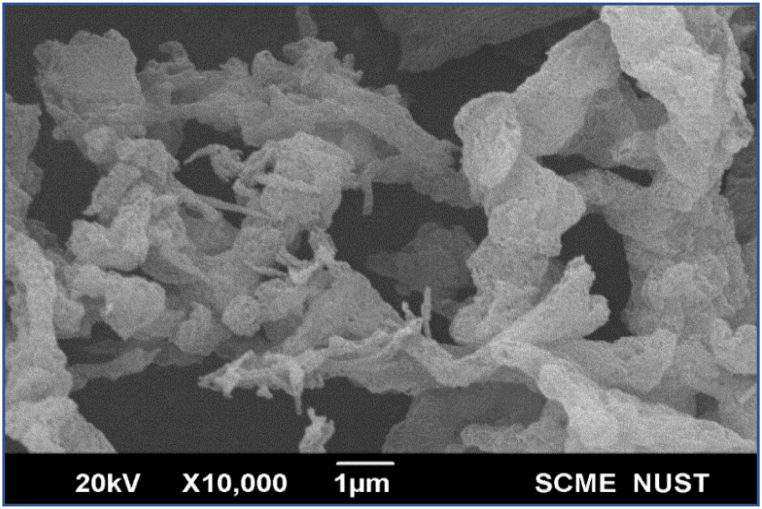
SEM image of g-C_3_N_4_.

Scanning Electron Microscopy is the emerging technology used for evaluating the specimen topography. It gives useful information regarding the material's shape and size. The SEM of g-C_3_N_4_ is shown in Fig. 4. Its ultrafine structure has also been established [50,51].The g-C_3_N_4_ single layers that are aligned with one another make up the material's structure. It has been demonstrated to include fluffy microspheres, which are made up of 2D-connected structure. Each sphere contains a good number of pores and voids [52]. Although the g-C_3_N_4_ may decompose into clusters or oligomers of condensed triazine-rings after being hydrothermally treated with sulfuric acid, some of these will be removed by hydrothermal decomposition because of their high water solubility, that leads to the development of porous network like structure in the layered g-C_3_N_4_. SEM provides useful information regarding the size and structure of g-C_3_N_4_ confirming its suitability to the drilling fluid applications for the shale drilling. First, the nanoscale size of the g-C_3_N_4_ as determined by SEM corresponds to the pore size of the shale, hence, nanoscale g-C_3_N_4_ can snugly fit into the shale pores plugging them and minimizing the fluid invasion deeper in the shale. This reduces the tendency of shale hydration and subsequently minimizes its swelling. Furthermore, the porous structure of g-C_3_N_4_ as determined by SEM makes it compressible and more resilient, hence, under hydrostatic pressure, the g-C_3_N_4_ particles compress and easily pass into the shale pores. While entering the shale pores, as the effect of hydrostatic head is reduced, the g-C_3_N_4_ particles regain the size and fit into the shale pores generating an effect called stress caging resulting in further stabilization of the shale [53].

### 3.4 Development of a g-C_3_N_4_-based water-based drilling fluid

The designed drilling fluid system contains several drilling fluid components as listed in Table 3. All these components are used to provide basic rheology and fluidity characteristics to the fluid to evaluate the true impact of g-C_3_N_4_ on the characteristics of drilling fluids. We used the same formulation with 0.5, 1 and 2 g of g-C_3_N_4_ to compare the results. To determine the real effect of NPs in drilling fluids, low Mud Weight (MW) drilling fluid of 10 lb/gal was used with various concentrations of nanomaterials. We developed four different formulations; the base drilling fluid comprises all the chemicals listed in Table 3 without nanomaterial; the other three formulations contain 0.5 g of g-C_3_N_4_ nanomaterial (0.0012 wt%), 1 g of g-C_3_N_4_ nanomaterial (0.0024 wt%), and 2 g of g-C_3_N_4_ nanomaterial (0.0048 wt%).

**Table 3:** The designed base fluid Formulation with addition of g-C_3_N_4_ nanomaterials.

Sr. No.	Component	Function	Specific Gravity	Concentration (g/350 mL)	Equivalent Volume (mL)
1	Water	Base Fluid	1.00	318.59	318.59
2	Soda Ash	Hardness Controller	2.53	0.10	0.04
3	Sodium hydroxide	Alkalinity Source	2.60	5.00	1.92
4	API Bentonite	Viscosifier	2.10	0.20	0.10
5	KCl	Shale Inhibitor	1.99	10.00	5.03
6	Polyanionic Cellulose	Fluid Loss Controller	1.50	2.00	1.33
7	Xanthan Gum Polymer	Rheology Modifier	1.50	1.00	0.67
8	Modified Starch	Fluid Loss Controller	1.50	2.00	1.33
9	g-C_3_N_4_ (Nano-Material)	Multiple Functions	2.33	0.5, 1, and 2 g	0.86
10	CaCO_3_-5 μm	Bridging Material	2.70	5.00	1.85
11	CaCO_3_-F (320 Mesh)	Bridging Material	2.70	5.00	1.85
12	API Barite	Weighing Agent	4.20	69.00	16.43
13	Total Volume				350 mL

### 3.5 Effect of synthesized g-C_3_N_4_ on the developed drilling fluid formulation's filtration and rheological characteristics

Table 4 shows the rheological and filtration properties of the produced drilling fluid. The experiments demonstrate an increasing trend in the drilling fluid rheological properties with the increasing concentration of g-C_3_N_4_. YP and AV have shown considerable improvement with the increasing g-C_3_N_4_ concentration. This increase in YP and AV is attributed to the improved hydrogen bonding and synergistic effect of the g-C_3_N_4_ and xanthan gum polymer. An increase in YP is desirable for effective hole cleaning during drilling operations. A reasonable range of AV is also necessary for the fluid consistency as it is subjected to varied shear rates in the wellbore during drilling and circulation [54,55]. A slight increase in PV was also observed with the increasing concentration of g-C_3_N_4_. The drilling fluid alkalinity value was maintained at pH 8.0 with the usage of potassium hydroxide for the optimum functioning of the polymers contained in the fluid formulation.

**Table 4:** Base drilling fluid and g-C_3_N_4_ treated fluid properties Drilling Fluid Hot Rolling conditions: Temperature: 225 °F Hot Rolling Time: 16 h.

Properties	Base Mud		0.0012 wt% g-C_3_N_4_		0.0024 wt% g-C_3_N_4_		0.0048 wt% g-C_3_N_4_	
	BHR	AHR	BHR	AHR	BHR	AHR	BHR	AHR
**MW (lb/gal)**	10	10	10	10	10	10	10	10
**PV (cP)**	16	7	16	10	17	11	21	19
**AV (cP)**	26	13	27	17	22	21	42	37
**YP (lb/100 ft**^**2**^**)**	20	12	24	14	27	20	42	36
**Gel’ (lb/100 ft**^**2**^**)**	7	5	7	6	9	8	15	13
**Gel**^**”**^**(lb/100 ft**^**2**^**)**	10	8	11	10	16	15	28	26
**API FL (mL)**	6	12.5	5.5	8.5	4.9	5.5	3.5	4
**Cake (mm)**	1.0	2.3	1.0	1.5	1.0	1.0	1.0	1.0
**pH**	8.0	8.0	8.0	8.0	8.0	8.0	8.0	8.0

#### 3.5.1 API filtrate

**Fig. 5:**
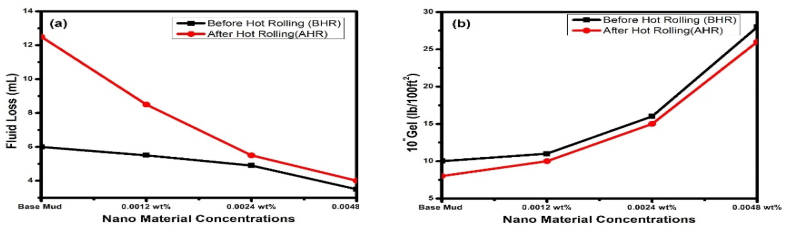
Effects of nanomaterials on **(a)** fluid loss **(b)** 10″ gel of base mud.

With the usage of an API filter press and conducting experiments at 100 psi pressure, filtration characteristics were assessed. Investigating filtration characteristics involved collecting information on factors including mud cake thickness after 30 min and filtration loss volume. After half an hour, the base mud sample's API fluid loss was 6.0 mL. Table 4 and Fig. 5a show the experimental findings as carried out with the API filter press. The use of g-C_3_N_4_ at various concentrations is found to gradually reduce the filtration loss volume up to 2.0 mL with 0.0048 wt% concentration BHR and very significant reduction in fluid loss was observed with g-C_3_N_4_ AHR. After hot rolling, the base mud experienced an extensive fluid loss of 12.5 mL. Nanomaterial considerably decreased this fluid loss, and as shown in Fig. 5a and Table-4, a fluid loss of just 4.0 mL was found with 0.0048 wt% g-C_3_N_4_ after hot rolling, the fluid loss controllers (Polyanionic Cellulose Low Viscosity and Modified Starch) partially lost their properties, causing substantial fluid loss in the base mud.

**Fig. 6:**
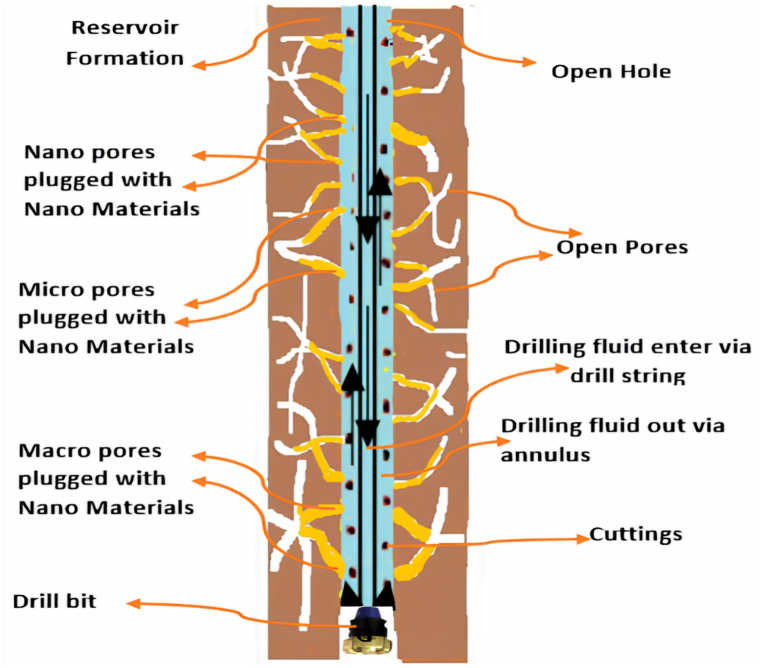
Graphical View of Shale plugging by nanomaterials to control Fluid Loss.

Fluid loss is the property, where nanomaterials play a critical role in minimizing its volume, as seen by the findings. The size of g-C_3_N_4_ is perfectly suited to plug the pore of an API filter paper. The g-C_3_N_4_ decreases the volume of filtration loss while maintaining a suitable suspended system and reducing flocculation of the particles by improving electrostatic interactions between g-C_3_N_4_ NPs and other particles. Deflocculated and suspended particles in a mud system may produce a thin, low-permeable mud cake on the surface of the lost region [56]. The electrostatic attraction between g-C_3_N_4_ and other solid particles, such as bentonite with a positive edge and a negative face, causes it to happen. A gelling structure might develop when g-C_3_N_4_ becomes attracted to other particles, enhancing mud consistency and lowering mud filtration volume. According to the results above, fluid loss is greatly reduced in 0.0024 wt% and 0.0048 wt% of g-C_3_N_4_ concentrations indicating a significant improvement in the fluid loss control. The fluid loss measured in the API filter press was reduced by 41.8 % and 68 % BHR and AHR, respectively, at 0.0048 wt% g-C_3_N_4_ concentration. Fig. 6 below, gives a graphical view of the plugging of shale pores by g-C_3_N_4_ and hence reducing the fluid loss.

#### 3.5.2 Gel strength

The Gel strength, which allows drill cuttings and mud particles to be suspended when the circulation stops during bottom hole assembly pick up or work over procedures, is the other important characteristic of mud rheology. The drilling fluid must have the capacity to carry drill cuttings while circulating and suspend the cutting while circulation is stopped. Table-4 displays the experimental findings from gel strength studies that were initially run for 10 s and afterwards for 10 min. The effect of NPs on gel strength at various concentrations in the range of 10 s to 10 min is shown in Fig. 5b. The nanomaterial-based drilling fluid formulations have good gel strength, the nanomaterial drilling fluid system's 10 s gel is 7, 9, and 15 lb/100 ft^2^ with 0.0012 wt%, 0.0024 wt%, and 0.0048 wt% g-C_3_N_4_ concentrations compared to base mud which have gel strength of 9 lb/100 ft^2^. The 10 min gel strength is 10, 11, 16 and 28 lb/100 ft^2^ for base mud, 0.0012 wt%, 0.0024 wt% and 0.0048 wt% g-C_3_N_4_ concentrations respectively which is necessary for cuttings suspension within the borehole. From the above results, we can infer that the gel strength increases with increasing the concentration of nanomaterial in the base fluid.

#### 3.5.3 Yield Point

It is important to notice from the above tables that the YP and AV dramatically increased with the inclusion of nanomaterials. The effective removal of drill cuttings from downhole, often referred to as hole cleaning, is achieved with a good range of the YP characteristics [57]. To facilitate the drill cuttings and prevent adding excess pressure to the drilling mud pump, the YP must be of a suitable value throughout the drilling operation.

**Fig. 7:**
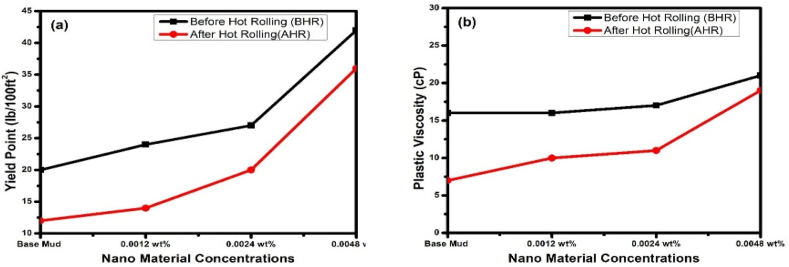
Effects of nanomaterials on (a) Yield Point and (b) Plastic Viscosity of base mud.

Due to the strong electrostatic attraction between the particles containing opposite charges, the drilling fluid's YP increases [56,58]. The YP of mud samples demonstrates varying efficacy at various nanomaterial concentrations. Base drilling fluid YP was recorded as 20 lb/100 ft^2^. The maximum drilling fluid YP level was 42 lb/100 ft^2^ at 0.0048 wt% of g-C_3_N_4_ concentration compared to based drilling fluid. Fig. 7a provides an illustration of how NPs affect the YP of drilling fluid samples. It has been noted through some earlier studies that NPs with higher surface areas per volume might enhance interactions with surrounding drilling fluid and the matrix. Previous studies have shown that a number of variables, including temperature and pH, might have a considerable impact on the morphology of NPs [59].

#### 3.5.4 Plastic Viscosity

Base drilling fluid PV was obtained as 16 cP, as shown in Fig. 7b. PV increases as friction between NPs and other mud system constituents increases. The PV value, however, varied for each concentration of NPs at different weight percent fractions, from 0.0012 to 0.0048 wt%. It is to be highlighted that viscosity is measured as internal friction in liquid layers, which is one reason why the presence of NPs causes the viscosity of mud to increase. So, the increase of viscosity may be caused by NPs in liquid layers [60,61]. Mud viscosity behavior is greatly influenced by the shape, density, and heat-transfer properties of nanomaterials. The increase in PV has also been observed in previous studies by directly mixing NPs with other solid particles, mainly bentonite. The NPs and bentonite mud may be joined through certain intermediary chemical connections in order to enhance the PV of the mud [62].

#### 3.5.5 Shale recovery test

To determine how varied concentrations of the synthesized nanomaterials influenced shale recovery ability during hot rolling shale dispersion studies, the shale samples were ground and sieved using 20–30 mesh size screens. 15 g of the cuttings were combined in an ageing cell to make 5 % KCl solution for hot rolling at 225° for 16 h. Only 88 % of the recovered shale in 5 % KCl solution indicated that the shale sample was extremely reactive. However, much higher shale recovery was obtained for solutions containing 0.0024 wt% and 0.0048 wt% g-C_3_N_4_ as 98.8 % and 99.6 %, respectively. It has been determined that NPs showed effective shale inhibitory properties.

#### 3.5.6 High pressure and high temperature filtration test

**Fig. 8:**
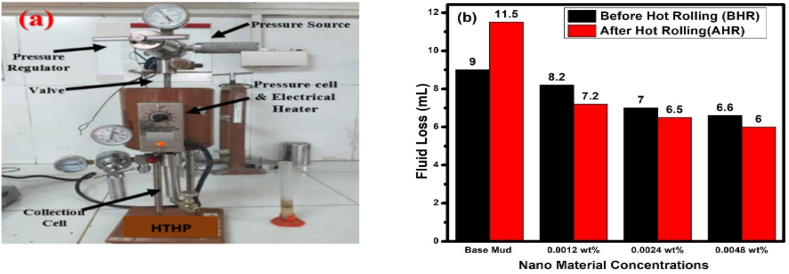
**(a)** HPHT Filter Press Equipment used (**b)** HPHT Fluid Loss BHR and AHR of base fluid and NPs treated base fluid.

A high-pressure testing for the loss of fluid during static filtration at high temperature was done in line with API standards. The testing system for HPHT filtration is shown in Fig. 8a. As shown in Fig. 8b and Table-5, distinct drilling fluid combinations were created using four different weight percentages of NPs in water-based drilling fluids. HPHT filtration test was run at 500 psi differential pressure and 225 °F temperature.

**Table 5:** HPHT Filtration Test Results of drilling fluid formulations at BHR and AHR Conditions.

Fluid types	Filtrate Volume (V_30_) BHR (mL)	Filtrate Volume (V_30_) AHR (mL)
Base fluid	9	11.5
Base fluid + 0.0012 wt% g-C_3_N_4_	8.2	7.2
Base fluid + 0.0024 wt% g-C_3_N_4_	7	6.5
Base fluid + 0.0048 wt% g-C_3_N_4_	6.6	6

The above data clearly show that 0.0048 wt% g-C_3_N_4_ treated fluid has the lowest fluid loss BHR and AHR when compared to the other three formulations and the base fluid. It is also noted that the nanomaterials-based drilling fluid after hot rolling greatly reduced fluid loss when compared to base fluid, which is due to the reason that after hot rolling, dispersion of NPs significantly enhanced and block the pores of filter paper, as well as agglomeration of g-C_3_N_4_ decreases. The fluid loss in base drilling fluid rises because the fluid loss inhibitors Polyanionic Cellulose Low Viscosity and modified starch partially decompose at high temperatures.

#### 3.5.7 Permeability plugging test

**Fig. 9:**
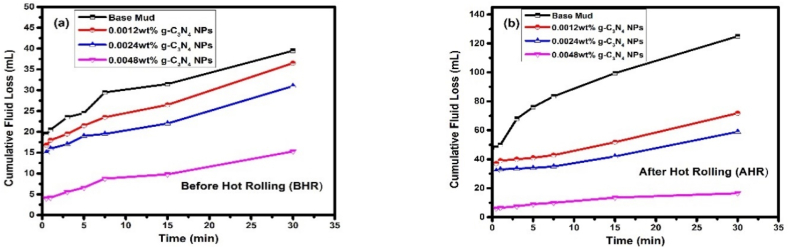
(a) PPT fluid loss BHR (b) PPT fluid loss AHR.

The OFITE Permeability Plugging Tester (PPT), which could replicate downhole static filtration utilizing a particular permeability disc as a filtering medium, was used to assess the plugging performance of g-C_3_N_4_. The PPT is modified version of a 500 mL HPHT Filter Press. The equipment is used to perform filtering tests on plugging materials without interference from particles that settle on the filter media. The differential pressures are generally set significantly greater than in typical HPHT filtration tests. The disc used in this investigation had a pore size 40 μm, the environment was 225°, 1000 psi of differential pressure, and the filtering process took 30 min [63,64]. Table-6 and Fig. 9a and b demonstrate the PPT fluid loss at 1000 psi differential pressure and 225° with g-C_3_N_4_ added to the base fluid. The fluid loss of the base fluid without the nano plugging agent was 39.5 mL before ageing and 125 mL after ageing. Compared to the base fluid, 0.0012 wt%, 0.0024 wt%, and 0.0048 wt% g-C_3_N_4_ treated fluids showed the much-improved plugging effect, reducing fluid loss to 36.5, 31, 15.3 mL before ageing and 71.8, 59, and 16.5 mL after ageing respectively. According to the PPT test, g-C_3_N_4_ has significantly minimized the fluid loss. High temperatures can cause g-C_3_N_4_ particles to disperse and slight reduction in agglomerate size. Under differential pressure, g-C_3_N_4_ particles can enter the nanopores and deform due to its resilient nature to produce dense plugging. While g-C_3_N_4_ particles can bridge in and provide a specific plugging effect in microscale pores. By following the two phenomena described above, g-C_3_N_4_ might drastically lower the rock's permeability and decrease PPT fluid loss. PPT abbreviation is used both to indicate the test and the testing equipment.

**Table 6:** Different drilling fluid formulations and their related BHR and AHR fluid loss after 30 min and corresponding spurt loss during PPT.

		Base Fluid		0.0012 wt% g-C_3_N_4_		0.0024 wt% g-C_3_N_4_		0.0048 wt% g-C_3_N_4_	
PPT @ 225 °F and 1000 psi ΔP	Units	BHR	AHR	BHR	AHR	BHR	AHR	BHR	AHR
**0.5 min**	mL	19.5	48	16.8	37	15	32	4	6
**1 min**	mL	1	2	1.2	2	1	1	0.2	0.5
**3 min**	mL	3	18	1.5	1	1	0.5	1.4	1
**5 min**	mL	1	8	2	1	2	0.5	1	1.5
**7.5 min**	mL	5	7.5	2	1.8	0.5	1	2.2	1
**15 min**	mL	2	16	3	9	2.5	7	1	3.5
**30 min**	mL	8	25.5	10	20	9	17	5.5	3
**Cumulative**	mL	39.5	125	36.5	71.8	31	59	15.3	16.5
**V**_PPT_	mL	79	250	81	143.6	62	118	30.6	33
**Spurt Loss**	mL	102	283	66	131.2	60	106	24.2	34
**Static Filtration Rate**	mL	5.84	18.62	10.22	14.60	6.57	12.41	4.02	2.19

According to the data in the table above, fluid loss decreases dramatically as nanomaterial concentration increases, with the best results provided by 0.0048 wt% g-C_3_N_4_, which is remarkable in comparison to other formulations. It is also important to note that the 0.0048 wt% g-C_3_N_4_ formulation has approximately same cumulated fluid loss before and after ageing.

#### 3.5.8 BET analysis to check the plugging of shale

The BET theory is the well-known model used to determine certain characteristics of the nanomaterials including the specific surface area, porosity volume, mean pore diameter and also used for calculating the pore size distribution of materials. In order to determine these parameters an adsorption rate is established by quantifying the adsorption of gas molecules on a given surface. According to the BET theory, the adsorption rate is directly proportional to the specific surface area of the analyzed material [65]. BET analyses have been used for different applications such as material studies, shale plugging, catalysis, concrete studies and membrane technology. Shale surface area and pore volume are evaluated using BET analysis, and these characteristics used to determine if the substance being studied has entered the Nano-pores and microspores.

**Fig. 10:**
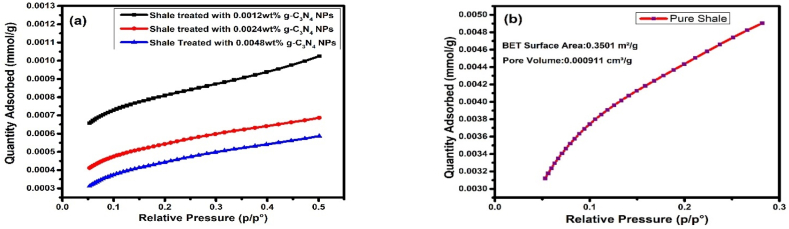
BET isotherm plots for nitrogen adsorption capability of (a) treated shale with.

Thus, BET studies were used to evaluate the plugging effect of g-C_3_N_4_ on selected shale samples. The results as shown in Fig. 10a and b depict that pure shale exhibits a high concentration of N_2_ gas adsorption in mmole/g of shale samples, indicating larger pore sizes and more pores width. On the other hand, when these shale samples are treated with varying concentrations of g-C_3_N_4_, there is a significant reduction in the amount of N_2_ gas adsorption, indicating the plugging of shales with g-C_3_N_4_ and decreased N_2_ gas adsorption capacity, pore size, and pore width upon increasing concentration.

In comparison to pure shale, which had BET surface area and pore volume of 0.3501 m^2^/g and 0.000911 cm³/g, respectively, treated shales had BET surface area and pore volume of 0.0583 m^2^/g and 0.000044 cm³/g; 0.0425 m^2^/g and 0.000032 cm³/g; and 0.0405 m^2^/g and 0.000029 cm³/g with 0.0012 wt%, 0.0024 wt%, and 0.0048 wt% of g-C_3_N_4_, respectively. From the above results, we conclude that the BET surface area and pore volume decrease with increasing the concentration of g-C_3_N_4_. Considerable decrease in surface area and pore volume was observed when pure shale was treated with 0.0012 wt% g-C_3_N_4_; when further increase in concentration was carried out, decrease in BET surface area and pore volume was observed, but comparatively lesser change was seen especially when the concentration was increased from 0.0024 wt% to 0.0048 wt%.

As depicted in Table-7, the pure shale surface area and pore volume were both comparatively large. The shale surface area and pore volume significantly decreased following treatment with 0.0012 wt%, 0.0024 wt%, 0.0048 wt% of g-C_3_N_4_, indicating that the shale's nano-pores have been partially filled and that pores and cracks have resulted in dense physical adsorption and filling. In practical situation, this would decrease the permeability of the borehole and the transmission of pore pressure. Thus, by physically blocking the shale surface, g-C_3_N_4_ reduces water infiltration, preventing water and shale from interacting and enhancing shale stability. The BET surface area and pore volume of treated shales were considerably lower than those of pure shales, indicating a considerable plugging of the nano-pores and micro-pores by g-C_3_N_4_.

**Table 7:** Comparative study of BET and Langmuir Surface area, Pore volume and pore width of pure shale and shales treated with different concentrations of g-C_3_N_4_.

Parameters	Pure Shale	0.0012 wt% g-C_3_N_4_	0.0024 wt% g-C_3_N_4_	0.0048 wt% g-C_3_N_4_
BET surface area (m^2^/g)	0.3501	0.0583 m^2^/g	0.0425	0.0405
Langmuir Surface Area (m^2^/g)	0.5613	0.0881	0.06680	0.0510
Pore Volume (cm^3^/g)	0.000911	0.000044	0.000032	0.000029
Pore Width (nm)	77.53	28.19	24.01	22.34

### 3.6 Dispersion and colloidal stability

Colloidally stable fluids are fully dispersed deflocculated fluids which can maintain their state for extended time periods. A colloidally stable drilling fluid depicts optimum fluid loss and cake thickness properties. Once a colloidally stable drilling fluid is flocculated, it shows a remarkable increase in the Fluid Loss. In a flocculated fluid, the colloidal particles aggregate or cluster, this increases the filter cake porosity and permeability which allows more filtrate to pass through it hence increasing the fluid loss. Conversely in a well dispersed fluid, colloidal particles are in a uniform distribution in the filter cake which reduces the cake permeability hence reducing the filtration volume. In a reference study, Peng et al. compared the Fluid Loss of deflocculated and flocculated drilling fluid and found that the Fluid Loss of the flocculated drilling fluid was 36 % higher than the deflocculated fluid [66]. This indicates that as the colloidal particles in a fluid agglomerate and fluid loses the colloidal stability, its fluid loss properties deteriorate by increasing in value to great extent.

Based on this study as we compare the same parameters of our fluid under study with and without the added g-C_3_N_4_ we find that the treated fluid did not show an increased in the Fluid Loss volume or cake thickness rather the Fluid Loss value as well as the cake thickness values reduced indicating a high level of colloidal stability in the treated fluid compared to the base fluid. We have extended the study to HPHT Fluid Loss and found the similar results. Hence the Fluid Loss of fluid treated with 0.0048 wt% of g-C_3_N_4_ was reduced to 4.0 mL/30min compared to the base fluid's Fluid Loss of 12.5 mL/30min and cake thickness reduced to 1 mm compared to base fluid cake thickness of 2.3 mm. HPHT Fluid Loss of the similar fluid was reduced from 11.5 mL/30min to 6.0 mL/30min; all values reported of the samples AHR at 225 °F for 16 h. These values indicate a good colloidal stability of the base fluid after treated it with the 0.0048 wt% of g-C_3_N_4_, the maximum concentration used in the study.

### 3.7 Limitations of the study and future research scope

Under the scope of present study, the application of g-C_3_N_4_ was limited to a WBM of 10 lb/gal which was hot rolled to 225 °F for 16 h. Within the limit of these parameters the study has shown promising results. However, considering the importance of g-C_3_N_4_ for drilling shales in trouble free manner in deeper horizons, the future studies can be conducted by extending these boundaries. First, as the thermal stability of g-C_3_N_4_ is quite high, in further studies the similar experiments can be performed at higher temperature such as 250 °F. This will enhance the application of g-C_3_N_4_ treated fluids to deeper wells where the downhole temperature increases. Second, the study can be extended to develop certain hybrids of g-C_3_N_4_ with such materials as nano-Silica and nano-Zirconium Oxide to carry out similar studies. Third, similar studies can be conducted to extend the application of g-C_3_N_4_ to Oil and Synthetic Based Mud Systems.

## 4 Conclusion

The g-C_3_N_4_ nanosheets were developed and used to improve the characteristics of water-based drilling muds. g-C_3_N_4_ is potential candidate as a chemical component in the formulation of water-based mud systems, based on the rheology, density, and filtration investigations. The findings of this investigation revealed that the addition of g-C_3_N_4_ had no impact on mud weight. The addition of 0.0048 wt% g-C_3_N_4_ to drilling mud increases its YP by 52 % and 66 % BHR and AHR respectively. A similar impact may be seen in the fluid loss measurement in PPT, where g-C_3_N_4_ decreased fluid loss by 61 % and 86 % BHR and AHR respectively with maximum shale recovery 99.8 %. We also discovered that the texture of the mud filter cake with added g-C_3_N_4_ is thinner and smooth compared to the filter cake obtained with base mud. Treated shales had BET surface area and pore volume of 0.0583 m^2^/g and 0.000044 cm³/g; 0.0425 m^2^/g and 0.000032 cm³/g; and 0.0405 m^2^/g and 0.000029 cm³/g with 0.0012 wt%, 0.0024 wt%, and 0.0048 wt% of g-C_3_N_4_, respectively, in contrast to pure shales, which had BET surface area and pore volume of 0.3501 m^2^/g and 0.000911 cm³/g, respectively. The g-C_3_N_4_ decreases water infiltration by blocking the shale surface, which inhibits water and shale from interacting and improves shale stability. These nanomaterials will be used in future to improve the performance of water-based drilling fluids at high temperatures and pressures conditions to drill longer shale sections.

## CRediT authorship contribution statement

**Anwar Ahmed:** Writing – original draft, Methodology, Formal analysis, Conceptualization. **Erum Pervaiz:** Supervision. **Iftikhar Ahmed:** Validation. **Tayyaba Noor:** Supervision.

## Data availability statement

Data will be made available on request.

## Declaration of competing interest

The authors declare that they have no known competing financial interests or personal relationships that could have appeared to influence the work reported in this paper.
